# The Role of Aggressive Peer Norms in Elementary School Children’s Perceptions of Classroom Peer Climate and School Adjustment

**DOI:** 10.1007/s10964-021-01432-0

**Published:** 2021-04-17

**Authors:** Lydia Laninga-Wijnen, Yvonne H. M. van den Berg, Tim Mainhard, Antonius H. N. Cillessen

**Affiliations:** 1 University of Groningen, Groningen, The Netherlands; 2 Utrecht University, Utrecht, The Netherlands; 3 Radboud University, Nijmegen, The Netherlands

**Keywords:** Aggressive peer norms, Classroom climate, School adjustment, Popularity, Victimization

## Abstract

Although prior research has indicated that peer norms for aggression enhance the spread of aggression in classrooms, it is unclear to date how these norms relate to students’ classroom climate perceptions and school adjustment. Aggressive descriptive norms reflect the average aggression of all students in classrooms, whereas aggressive popularity norms represent the extent to which aggressive behavior relates to popularity among peers. This study examined the role of aggressive descriptive and popularity norms in the classroom climate perceptions (cooperation, conflict, cohesion, isolation) and school adjustment (feelings of belonging; social, academic, and general self-esteem) of popular, well-liked, and victimized children. Self-reported and peer-nominated data were obtained from 1511 children (*M*_age_ = 10.60 years, SD = 0.50; 47.2% girls) from 58 fifth-grade classrooms. The results indicated that aggressive descriptive and popularity norms both matter in elementary school, but in diverging ways. Specifically, aggressive descriptive norms—rather than popularity norms—contributed to negative classroom climate perceptions irrespective of students’ social position. In addition, whereas descriptive norms contributed to between-classroom variations in some aspects of school adjustment, aggressive popularity norms related to increased school maladjustment for popular and victimized children specifically. Thus, aggressive descriptive norms and popularity norms matter in complementary ways for children’s classroom climate perceptions and adjustment in elementary education.

## Introduction

In many countries, schools have the legal responsibility to formulate protocols that ensure school safety, promote a positive classroom peer climate, and foster students’ school adjustment. Schools are carefully monitored on these indicators of school quality by the inspection of education (Orobio de Castro et al., 2018). One factor that may hinder schools in achieving their protocol goals, are peer norms for aggression. Peer norms reflect a consensus on the behaviors that are expected and seen as appropriate by peers in a classroom (Shaw, 1981). Peer norms often are assessed with descriptive norms, reflecting the average behavior of all students in a classroom, not distinguishing between more and less influential peers. More recently, it has been argued that popular peers in particular function as role models and set a norm (“popularity norm”) for the behaviors that are considered desirable and valuable in the classroom (Dijkstra & Gest, 2015). To date, norm research has primarily focused on secondary schools, and mostly examined the relative role of descriptive and popularity norms on adolescents’ behavioral and relational choices (e.g., Laninga-Wijnen et al., 2017). This work has shown that aggressive popularity norms rather than descriptive norms strengthen the acceptance and spread of aggression in classrooms (Dijkstra & Gest, 2015; Laninga-Wijnen et al., 2020). Given that perpetrating aggression, or being confronted with it, is a strong predictor of students’ social, psychological, and academic problems (Goldstein et al., 2008), it is likely that classrooms with aggressive peer norms are characterized by a more negative peer climate and school maladjustment among students. Yet to date, there have been no efforts to parse out the differential role of aggressive popularity and descriptive norms in these indicators of classroom and student functioning. Moreover, it can be hypothesized that students’ social position in the classroom (e.g., being victimized, popular, or well-liked) either increases or mitigates harmful effects of aggressive peer norms on their perceptions of the classroom climate and school adjustment. Therefore, this study aimed at identifying the role of both aggressive descriptive and popularity norms in the classroom climate perceptions and school adjustment of victimized, popular, and well-liked students.

### Aggressive Peer Norms, Classroom Climate Perceptions, and School Adjustment

The classroom climate entails several components, such as how children perceive the interactions between their classmates (the degree of cooperation, conflict) and the within-classroom structure of peer relationships (the degree of cohesion, isolation; Boor-Klip et al., 2016). Although it speaks to intuition that aggressive descriptive and—perhaps in particular—popularity norms would create an undesirable classroom climate, there is surprisingly little research on this topic. Only few studies have shown that when children are confronted with much aggression in their classroom (high aggressive descriptive norm) they perceive their classroom as unsafe and full of conflict (Goldstein et al., 2008; Koth et al., 2008). Furthermore, descriptive and popularity norms of aggression have been linked with more rejection and diminished caring for each other (Dijkstra et al., 2008; Gilman et al., 2009). Thus, higher aggressive norms may make children perceive lower cooperation and cohesion, and more isolation and conflict.

Aggressive norms may also relate to children’s school adjustment, including their general, social, and academic self-esteem as well as feelings of connectedness with classmates. It can be theorized that in classrooms where aggression is normative, students have fewer opportunities to engage in positive, trustful peer relationships and rather try to avoid becoming the next victims (Saarento et al., 2015). This hinders the development of social skills and decreases feelings of connectedness with classmates. In line with this reasoning, descriptive norms for aggression were found to increase loneliness and popularity norms were linked to decreased school wellbeing (Bellmore et al., 2004; Dijkstra & Gest, 2015). Moreover, when aggression is normative, students may pay more attention to aggressive acts, which distracts them from learning and makes them attach less value to doing well academically (Thomas et al., 2011). A few studies found that aggressive descriptive norms related to lower academic involvement (Wang et al., 2019) and that aggressive popularity norms related to lower academic achievement (Dijkstra & Gest, 2015). Thus, aggressive descriptive and popularity norms may decrease feelings of belongingness and reduce social, general, and academic self-esteem.

Although prior work provided valuable insights in the role of aggressive norms in some aspects of the classroom climate and school adjustment, several gaps remain that are addressed in the current study. First, the potentially differential role of descriptive versus popularity norms in classroom climate perceptions and adjustment remains unknown to date. Whereas in the past, most attention has been paid to descriptive norms, recently popularity norms gained increasing attention (Laninga-Wijnen & Veenstra, 2021), and research demonstrated that popularity norms rather than descriptive norms predicted adolescents’ social and behavioral development (Laninga-Wijnen et al., 2020). This is in line with social impact theory (Latané, 1981), which states that even a minority of people can exert strong influence on social groups if others in the group are dependent on them, for instance because this minority has a powerful status. Being popular among one’s peers can be an indicator of such powerful status, and hence, in particular popular peers may set a norm in classrooms for which behaviors are considered valuable or important. Moreover, popular peers are often more visible and central in the peer network. Thus, when they display aggression, students are more likely to notice this. Therefore, popularity norms may play a more important role than descriptive norms in explaining variations between classrooms in students’ classroom climate perceptions and school adjustment, yet this has not been tested to date. Thus, the current study will be the first to disentangle the role of descriptive and popularity norms in students’ classroom climate perceptions and adjustment.

A second way in which this study extends prior work is by examining the role of descriptive and popularity norms in late childhood rather than in adolescence, which is interesting from a developmental perspective. From late childhood onwards, the desire for popularity increases and displaying aggression becomes an increasingly important way through which a popular position can be achieved or maintained (Cillessen & Mayeux, 2004; Dawes & Xie, 2017). Therefore, the importance of aggressive popularity norms for both classroom and individual functioning may already start at the end of elementary school. Examining norms in elementary school is also interesting from an educational perspective. Unlike middle school or high school, students in elementary schools spend all of their lessons within the same classroom, which potentially exacerbates the importance of classroom norms. It is hypothesized that both popularity and descriptive norms contribute to more negative classroom climate perceptions and maladjustment (Hypothesis 1a), but that popularity norms play a stronger role than descriptive norms (Hypothesis 1b). The latter hypothesis is stated with caution, as this study is the first to examine popularity norms in elementary school.

### Aggressive Norms and Classroom Perceptions and Adjustment of Victimized, Popular, and Well-liked Students

A third way in which this study extends upon prior work is by examining whether the role of norms varies for different types of students. Not every individual may be equally affected by the classroom norm. Specifically, potential harmful effects of aggressive norms may depend on students’ social position in their classroom (being victimized, popular, or well-liked), which is described into more detail in the paragraphs that follow.

#### Aggressive norms and victimized children

Being a victim of bullying takes a large social and emotional toll. Victims perceive their classroom as more negative and are at higher risk of social-emotional and academic problems than non-victimized students (Arseneault, 2018; Boor-Klip et al., 2017). Yet, victims’ classroom perceptions and adjustment may vary across classrooms, depending on peer norms for aggression. Moreover, descriptive norms and popularity norms for aggression may relate to victims’ adjustment in diverging ways.

Regarding descriptive norms, perhaps somewhat counterintuitively, it can be reasoned that victims do better in classrooms with higher aggressive descriptive norms. This reasoning is consistent with a phenomenon recently illuminated in the literature: the healthy context paradox, which—paradoxically—indicates that victims are better adjusted in more negative contexts. Victims had less social anxiety (Bellmore et al., 2004) and more perceived social competence (Morrow et al., 2019a) in classrooms with higher aggressive descriptive norms. Victims also had higher self-esteem and lower depressive symptoms in classrooms with more victimization (Huitsing et al., 2019). This healthy context paradox can be explained based on attribution theory. Victims make attributions to explain why they are being victimized, including locus (whether the cause of victimization is internal or external to the victim) and controllability (whether the cause can be changed; Morrow et al., 2019b). Especially internal attributions put victims at risk for psychosocial problems (Schacter & Juvonen, 2015). In classrooms where aggression is normative, victims may be less likely to blame themselves and make internal attributions, but rather attribute the victimization to external causes. It therefore was hypothesized that victims have more positive classroom climate perceptions and better school adjustment when descriptive norms for aggression are higher (Hypothesis 2a).

It is unlikely, however, that popularity norms for aggression would generate a similar healthy context paradox. As mentioned before, aggressive popularity norms reflect how valuable or important a certain behavior is, rather than how common it is. As such, popularity norms may not help victims to refrain from making internal attributions for their victimization. Instead, aggressive popularity norms may pose an additional risk to victims. When aggression is endorsed by popular peers, students tend to be reluctant to behave against this norm nor do they feel the urge to intervene in bullying (Peets et al., 2015). This possibly enhances victims’ feeling that nobody cares about them (Schacter & Juvonen, 2018). Consequently, victims may feel worse when aggressive popularity norms are higher (Hypothesis 2b).

#### Aggressive norms and popular children

Popularity is a social reputation characterized by social power, centrality, and visibility (Cillessen & Marks, 2011). Popular students possess characteristics that are valued by the peer group (e.g., being attractive, athletic, and well-known; Cillessen & Van den Berg, 2012). Being in such a powerful, admired position may generally provide popular students with positive classroom experiences and self-confidence (Anderson et al., 2015). Nevertheless, popular students’ adjustment may vary across classrooms depending on the aggressive norms, and it again can be expected that descriptive and popularity norms work out in diverging ways.

Regarding descriptive norms for aggression, it can be reasoned that popular children are less likely to be targets of aggression as peers may know that they have the social and material resources to fight back (Hawley & Bower, 2018). Thus, popular children may benefit from their status, irrespective of the aggressive descriptive norm (Hypothesis 3a).

However, popularity norms for aggression may work out negatively for popular students. In classrooms with aggressive popularity norms, it is likely that popular students are viewed as more aggressive among their classmates. Therefore, classmates may not want to befriend popular peers but rather avoid and dislike them (Juvonen et al., 2003). This impairs popular students’ ability to develop close and intimate relationships at school, which may adversely relate to their classroom climate perceptions and school adjustment. Accordingly, a recent study found that highly popular adolescents were “lonely at the top” and had low social self-esteem (Ferguson & Ryan, 2019). Furthermore, aggressive popular students are more likely to fail academically than non-aggressive popular students (Schwartz et al., 2006). Therefore, popular children can be hypothesized to have less positive classroom experiences and lower school adjustment when aggressive popularity norms are higher (Hypothesis 3b).

#### Aggressive norms and well-liked children

Traditionally, well-liked students are considered to flourish academically, socially, and emotionally (Ryan & Shin, 2018), but it is unknown whether their adjustment varies across classrooms depending on the aggressive norm. Well-liked students are frequently preferred over other students as activity partners, academic helpers, and friends (Ryan & Shin, 2018). They have high-quality friendships at school and do well academically (Cillessen & Van den Berg, 2012). Well-liked children may thus benefit from their social skills and large, high-quality social network that provides support and that potentially shields them from the detrimental role of aggressive descriptive and popularity norms (Cullum et al., 2013). However, well-accepted children are also higher on empathy and may be more attuned to other students’ wellbeing and functioning (Oberle et al., 2010). They may have a more realistic picture of the impact of aggressive norms on classmates’ wellbeing. As a consequence, they may perceive their classroom as more negative and feel less well at school when aggression is normative. Therefore, the role of aggressive descriptive and popularity norms on well-liked students’ perception of the classroom climate and school adjustment will be explored.

## Current Study

Prior research has indicated that aggressive peer norms enhance the acceptance and proliferation of aggression (Laninga-Wijnen et al., 2017), but it is unknown how these norms relate to students’ classroom climate perceptions and school adjustment. Moreover, norm research to date primarily focused on secondary school students, and hardly considered whether certain students may be more strongly affected by these norms than others. The current study therefore examined the relative contribution of aggressive descriptive and popularity norms in students’ classroom climate perceptions and school adjustment in an elementary school sample, and tested whether the role of norms varied depending on students’ social standing (victimized, popular, and well-liked). It was hypothesized that classrooms with higher aggressive descriptive and popularity norms are characterized by a more negative classroom climate and lower adjustment among students (Hypothesis 1a). It was also expected that popularity norms more strongly predict these classroom-level outcomes than descriptive norms (Hypothesis 1b), because the visible position of popular peers makes it more likely that their aggression is being noticed and impactful. Yet, this hypothesis was formulated with caution as all prior work on popularity norms was conducted in secondary schools (e.g., Laninga-Wijnen et al., 2020).

Furthermore, it was tested whether aggressive norms work out differently for victims, popular, and well-liked students. Based on recent work illuminating a healthy context paradox (Huitsing et al., 2019), it was hypothesized that victims may have more positive classroom climate perceptions and better adjustment when aggressive descriptive norms are higher (Hypothesis 2a). Instead, higher aggressive popularity norms may pose an additional risk to victims because others are less likely to intervene when aggression is seen in a positive light (Peets et al., 2015; Hypothesis 2b). Aggressive descriptive norms may be unimportant to classroom climate perceptions and adjustment of popular children, because they are less likely to be targeted (Hypothesis 3a). Popular children may have less favorable classroom climate perceptions and adjustment in classrooms with higher aggressive popularity norms because other peers may avoid and dislike them (Hypothesis 3b). It was also explored whether the classroom climate perceptions and school adjustment of well-liked students varied as a function of popularity and descriptive norms. Gender, age, and aggressive behavior were included as covariates as they are known to relate to adjustment and classroom climate perceptions (Laninga-Wijnen et al., 2021).

## Method

### Participants

The data for this study came from a larger project on classroom climates that was conducted in 58 fifth-grade classrooms of 40 elementary schools in The Netherlands (Peer Climate Study). It was an intervention study aimed at improving the classroom climate. Data for the current study represented the pre-intervention assessment (e.g., first wave of the project), after which classrooms were randomly assigned to the intervention or control condition. Thus, the current data were not influenced by the intervention. Students’ mean age was 10.60 years (SD = 0.50) and 47.2% were girls. Based on the classification by Statistics Netherlands (CBS, 2021), 83.0% of the students were Dutch (i.e., both parents born in The Netherlands), which was representative for the areas in which the schools were located. Only students for whom informed parental consent was obtained, participated (1511 out of 1533; participation rate 98.6%).

### Procedure

Schools were contacted by telephone and letter in the Fall of 2012. After the principal and respective teacher(s) agreed to participate, parents received a letter explaining the goal of the study and requesting active informed consent. Children were asked for assent and completed the questionnaire on netbook computers. Confidentiality was emphasized during instructions of researchers and children were seated separately with partition screens on their desks. During administration, teachers worked at their desks in the classroom. A researcher was available to answer children’s questions. The study was approved by the ethics review board of the institute of the last author (Radboud University, Nijmegen, The Netherlands).

### Measures

Most predictor variables were based on peer nomination data. Participants indicated which of their classmates best fit a certain description. Students could nominate as many or as few classmates as they wanted and were also allowed to nominate nobody. To avoid sequence effects (Poulin & Dishion, 2008), the order of classmates’ names was randomized for each participant, yet remained the same across questions. For each question, the number of nominations received was divided by the number of potential nominators (i.e., number of classmates minus the number of children who were absent or did not have consent). The resulting scores reflect the proportion of classmates who nominated a participant for that item. Individual-level predictor variables were centered at the classroom mean.

### Individual-level Predictor Variables

#### Being well-liked

Being well-liked was derived from nominations for the question “Which classmates do you like most?” (cf. Hopmeyer Gorman et al., 2011). The peer acceptance score was the proportion of classmates who nominated a child as well-liked and could vary from 0 (not nominated by anyone) to 1 (nominated by all voters).

#### Popularity

Popularity was assessed by asking “Who is most popular?” and “Who is least popular?”. A composite score for popularity was computed by subtracting the least popular proportion score from the most popular proportion score (Cillessen & Marks, 2011; Van den Berg et al., 2020). This score could vary from −1 (nominated by all voters as least popular and by none as most popular) to 1 (nominated by all voters as most popular and by none as least popular).

#### Victimization

Peer victimization was based on a self-report measure (Olweus, 1996). Children rated the degree to which they were victimized by their peers on a 5-point Likert scale (1 = not at all; 5 = very much). As the variable was positively skewed, it was dichotomized by identifying students who scored a 4 (e.g., “much”) or 5 (e.g., “very much”) as victim (1) and students who scored below 4 as non-victim (0). The two upper categories were chosen as cut off point for being a victim, as victimization is usually defined as a relatively structural, stable, and severe experience (Solberg & Olweus, 2003). In this way, 83.8% of the participants were classified as non-victims and 16.2% as victims (cf. 10.9% Solberg & Olweus, 2003; 15% Olweus, 1996; about 17% Cowie & Olafsson, 2001).

### Individual-level Control Variables

#### Aggression

Aggression was measured with two peer nomination items for physical or verbal aggression (“Which classmates call other children names?” and “Which classmates hit or kick other children?”), two peer nomination items for relational aggression (“Which classmates gossip about other children?” and “Which classmates exclude other children?”), and one peer nomination item for bullying (“Which classmates bully other children?”). Principal component factor analyses indicated that these five items convincingly loaded on one factor (factor loadings varying from 0.67 to 0.95), explaining 75.5% of the variance. Reliability analyses indicated strong internal consistency, with *α* = 0.91. Therefore, the five proportion scores were averaged to create one scale for aggressive behavior. Scores could theoretically vary from 0 (not nominated on any of the five aggression items) to 1 (nominated by all voters on all five aggression items).

#### Teacher-assigned grades

In analyses predicting academic self-esteem, teacher-assigned average grades based on Math and Language was included as control variable for every student, as grade strongly relates to how students evaluate their academic functioning (Gest et al., 2008). Teachers graded students’ performance on a scale from 0 to 10 for each subject. It was not included in models with other outcome variables as there were no strong reasons to do that (correlations with teacher-assigned grade and other variables were low, see Table 1) and the aim was to keep other models parsimonious.

**Table 1:** Descriptives and correlations between unstandardized individual-level and classroom-level variables

	*M* (SD)	Range	1	2	3	4	5	6	7	8	9	10	11	12	13
*Individual-level variables*															
1. Victim	0.16 (0.37)	0.00–1.00													
2. Peer-perceived aggression	0.12 (0.14)	0.00–0.86	0.13***												
3. Well-liked	0.14 (0.09)	0.00–0.54	−0.17***	−0.25***											
4. Popularity	−0.02 (0.30)	−0.94–0.95	−0.23***	0.23***	0.35***										
5. Cooperation	3.84 (0.63)	1.00–5.00	−0.27***	−0.06*	0.13***	0.18***									
6. Conflict	2.75 (0.80)	1.00–5.00	0.34***	0.17***	−0.14***	−0.07**	−0.45***								
7. Cohesion	2.79 (0.80)	1.00–5.00	−0.18***	−0.08**	0.05*	0.04	0.55***	−0.46***							
8. Isolation	2.84 (0.82)	1.00–5.00	0.25***	0.13***	−0.08***	−0.003**	−0.39***	0.58***	−0.44***						
9. Belonging	4.22 (0.78)	1.00–5.00	−0.43***	−0.10***	0.19***	0.25***	0.61***	−0.42***	0.41***	−0.37***					
10. Academic self-esteem	3.54 (0.69)	1.00–5.00	−0.07**	−0.10***	0.06***	0.004	0.19***	−0.12***	0.10***	−0.12***	0.25***				
11. General self-esteem	4.03 (0.73)	1.17–5.00	−0.35***	−0.13***	0.16***	0.13***	0.36***	−0.27***	0.25***	−0.27***	0.58***	0.41***			
12. Social self-esteem	3.60 (0.77)	1.00–5.00	−0.40***	−0.09***	0.27***	0.43***	0.44***	−0.30***	0.27***	−0.25***	0.62***	0.29***	0.53***		
13. Age	10.60 (0.50)	8.43–12.79	0.01	0.13**	−0.09**	0.04	−0.002	0.03	0.04	0.09**	−0.02	−0.15***	−0.05*	−0.04	
14. Teacher-assigned grade	7.15 (1.36)	2.00–10.00	−0.11***	−0.17***	0.22***	0.10***	0.04	−0.07*	−0.08**	−0.01	0.12***	0.45***	0.16***	−0.14***	−0.30***
	*M* (SD)		1	2	3										
*Classroom-level variables*															
1. Aggressive popularity norm^a^	0.28 (0.34)	−0.41–1.20													
2. Aggressive descriptive norm	0.12 (0.04)	0.05–0.28	0.34**												
3. Classroom size	26.05 (4.25)	18–42	−0.12	−0.41**											

*p < 0.05; **p < 0.01; ***p < 0.001

^a^Fisher’s *z*-score correlation

### Classroom-level Predictor Variables

#### Descriptive norm

Aggressive descriptive norms were measured as the aggregated score for peer-nominated aggression across all classmates (cf. Laninga-Wijnen et al., 2017). As such, this measure represents a certain level of consensus among classmates on the extent to which they view each other as aggressive. This “consensus” is core to the definition of norms, and having multiple nominators enhances the validity and reliability of data (Bukowski et al., 1993). Scores were standardized and centered at the grand mean.

#### Popularity norm

This was calculated as the within-classroom correlation between peer-nominated popularity and aggression (cf. Gest & Rodkin, 2011). Correlations were transformed into Fisher *z*-scores, to obtain a relatively normally distributed measure [*z*′ = 0.5[ln(1 + *r*) – ln(1 – *r*)] (Fisher, 1925)], and centered at the grand mean.

#### Classroom size

Classroom size was a control variable in the analyses predicting cohesion, isolation, cooperation and conflict. Scores were *z*-standardized and centered at the grand mean.

### Individual-level Outcome Variables

#### Perception of classroom climate

Perceptions of classroom climate was assessed with four aspects of the Classroom Peer Context Questionnaire (CPCQ; Boor-Klip et al., 2016), namely cooperation (4 items), conflict (4 items), cohesion (3 items), and isolation (4 items). Example items are: “In this classroom, children help each other” (cooperation), “In this classroom, children argue with each other” (conflict), “In this classroom, everyone plays together during recess” (cohesion), and “In this classroom, some children do not belong to the group” (isolation). Children rated each item on a 5-point scale (1 = not true at all; 5 = completely true). For each scale, the average was calculated. Cronbach’s *α* was sufficient to good for all scales (0.79, 0.83, 0.68, and 0.74 for cooperation, conflict, cohesion, and isolation, respectively).

#### Students’ school adjustment

##### Feelings of belonging

The fifth scale of the CPCQ assessed whether students themselves felt connected to their classroom (Boor-Klip et al., 2016). The scale consisted of 4 items, such as “In this classroom, I can be myself”. The average was computed. Cronbach’s α was good (*α* = 0.83).

##### Self-esteem

Self-esteem was measured with the Dutch Version of the Harter Scales (CBSK; Veerman et al., 2004). The CBSK has 18 items to measures general self-esteem (6 items) academic self-esteem (6 items), social self-esteem (6 items). Example items were “I am content with the person who I am” (general self-esteem), “I am doing well at school” (academic self-esteem) and “My classmates like me” (social self-esteem). Children rated the degree to which each item was true for them on a 5-point scale (1 = not at all; 5 = very much). The internal consistency (Cronbach’s *α*) was 0.83 for general self-esteem, and 0.80 for social self-esteem. For these scales, the six items were averaged. For academic self-esteem, excluding one of the six items resulted in an increase of Cronbach’s α (from 0.74 to 0.76). Therefore, a scale was created based on the average of five items.

### Analysis Strategy

In total, 47 students (3.1%) were absent during data collection and therefore had missing data on self-reported variables. It was examined whether these partially missing cases differed from complete cases in popularity, being well-liked, victimization, ethnicity, and gender. There were no significant differences between partially missing cases and complete cases.

Multi-level regression analyses were conducted in Mplus Version 8 (Muthén & Muthén, 2016), with the MLR-estimator (Yuan & Bentler, 2000) to account for the potential non-normal distribution of the residuals. One model was tested for each outcome variable separately (four dimensions of classroom climate, feelings of belonging, and three types of self-esteem) to prevent that models included more parameters than data. As a preliminary step, empty models with intraclass correlations were examined. Next, “main models” were tested that included individual- and classroom-level predictors to test Hypothesis 1a that aggressive norms relate to more negative classroom climate perceptions and lower school adjustment. To determine whether popularity norms would play a stronger role in these outcomes (Hypothesis 1b), main models were run again for descriptive and popularity norms separately, to evaluate beta’s and to compare how much of the variance was explained by each of these norms separately.

Next, the potentially differential importance of descriptive and popularity norms for different types of students (victims, popular, and well-liked students) was examined. To this end, random slopes were included for the links between victimization, popularity, being well-liked, and the outcome variables. The slopes for the three types of social position were included simultaneously, to take potential covariation between these random slopes into account and to avoid testing the same model three times for each social position predictor - which reduces chance capitalization. Subsequently, cross-level interactions were tested to examine to what degree variability in the associations between students’ social position and classroom climate perceptions or school adjustment was explained by aggressive descriptive and popularity norms. The models containing cross-level interactions were only interpreted if they had a better fit compared to the main models, and if cross-level interactions were statistically significant. Model fit was assessed based on a decrease in AIC and BIC, where more weight was put on a decrease in AIC, as the BIC has been shown to be very conservative which may be problematic when power to detect effects is limited (LaHuis & Ferguson, 2009). Simple slopes analyses were conducted with the Preacher and Hayes method for multi-level analyses (Preacher et al., 2006) to interpret significant cross-level interactions.

## Results

### Descriptive Statistics

Table 1 provides the descriptive statistics including correlations of the main study variables. Aggressive popularity and descriptive norms varied strongly between classrooms. The correlation between aggressive popularity and descriptive norms (*r* = 0.34, *p* = 0.009) indicated a significant yet limited overlap. Victimization was significantly associated with maladjustment in all areas whereas being well-liked and being popular were both significantly associated with more positive classroom climate perceptions and adjustment. Intraclass correlations for social self-esteem, general self-esteem, academic self-esteem, and feelings of belonging were low (ICC_social_ = 0.021; ICC_academic_ = 0.019, ICC_general_ = 0.002; ICC_belonging_ = 0.033), indicating that at most 3% of the variability in students’ school adjustment was between classrooms. Intraclass correlations for isolation, cohesion, cooperation, and conflict were 0.095, 0.106, 0.086, and 0.169, respectively.

### Aggressive Norms and Classroom Climate Perceptions and School Adjustment

Main models including both individual- and classroom-level predictors were run to examine whether aggressive norms predict variations between classrooms in students’ classroom climate perceptions and school adjustment. In line with Hypothesis 1a, higher aggressive descriptive norms were associated with higher classroom levels of perceived conflict, more isolation, and lower cohesion and cooperation (Table 2). Higher aggressive descriptive norms also related to lower social self-esteem and lower feelings of belonging among students but were not associated with academic and general self-esteem (Table 3). In contrast to Hypothesis 1a, aggressive popularity norms did not predict classroom-level variations in students’ classroom climate perceptions or adjustment - except for isolation and conflict, but in an unexpected direction: higher aggressive popularity norms related to lower levels of perceived isolation and conflict.

**Table 2:** Main models examining the role of social reputation and aggressive peer norms in classroom climate perceptions

	Perceived cooperation				Perceived conflict				Perceived isolation				Perceived cohesion			
	*B*	SE	*p*	Beta	*B*	SE	*p*	Beta	*B*	SE	*p*	Beta	*B*	SE	*p*	Beta
*Individual-level*																
Gender (0 = boy)	0.011	0.040	0.790	0.009	−0.074	0.042	0.074	−0.051	0.042	0.052	0.435	0.027	0.080	0.049	0.104	0.053
Age	−0.002	0.031	0.947	−0.002	−0.003	0.036	0.931	−0.002	0.094	0.043	0.031	0.060	0.096	0.045	0.032	0.063
Victimization (0 = non-victim)	−0.377	0.054	<0.001	−0.225	0.585	0.055	<0.001	0.290	0.496	0.053	<0.001	0.230	−0.320	0.068	<0.001	−0.152
Popularity	0.219	0.058	<0.001	0.108	0.032	0.066	0.634	0.013	0.155	0.087	0.073	0.059	−0.005	0.084	0.952	−0.002
Liked	0.339	0.241	0.159	0.047	−0.507	0.267	0.058	−0.058	−0.408	0.285	0.152	−0.044	0.051	0.271	0.851	0.006
Peer-perceived aggression	0.019	0.159	0.904	0.004	0.282	0.173	0.103	0.053	0.363	0.141	0.010	0.064	−0.130	0.155	0.399	−0.023
*Classroom-level*																
Aggressive descriptive norm	−0.147	0.030	<0.001	−0.777	0.295	0.040	<0.001	0.887	0.163	0.035	<0.001	0.645	−0.158	0.044	<0.001	−0.600
Aggressive popularity norm	0.038	0.069	0.578	0.069	−0.274	0.087	0.002	−0.278	−0.203	0.096	0.034	−0.272	0.070	0.100	0.482	0.090
Classroom size	−0.014	0.006	0.015	−0.322	0.036	0.007	<0.001	0.462	0.029	0.006	<0.001	0.494	−0.026	0.007	<0.001	−0.416
Residual variance within	0.331	0.015	<0.001	0.917	0.466	0.016	<0.001	0.893	0.556	0.018	<0.001	0.932	0.550	0.024	<0.001	0.968
Residual variance between	0.018	0.005	0.001	0.526	0.043	0.011	<0.001	0.399	0.039	0.011	0.001	0.617	0.047	0.014	0.001	0.693
Variance explained within	0.083	0.016	<0.001		0.107	0.017	<0.001		0.068	0.013	<0.001		0.032		0.002	
Variance explained between	0.474	0.114	<0.001		0.601	0.093	<0.001		0.383	0.115	0.001		0.307		0.002	

*CI* Confidence interval

**Table 3:** Main models examining the role of social reputation and aggressive peer norms in students’ school adjustment

	Social self-esteem				General self-esteem				Academic self-esteem				Belonging			
	*B*	SE	*p*	Beta	*B*	SE	*p*	Beta	*B*	SE	*p*	Beta	*B*	SE	*p*	Beta
*Individual-level*																
Gender (0 = boy)	−0.055	0.039	0.153	−0.037	−0.172	0.046	<0.001	−0.118	−0.100	0.036	0.006	−0.073	−0.135	0.043	0.002	−0.088
Age	−0.050	0.037	0.175	−0.032	−0.054	0.039	0.171	−0.037	−0.009	0.037	0.806	−0.007	−0.017	0.037	0.637	−0.011
Victimization (0 = non-victim)	−0.614	0.056	<0.001	−0.291	−0.661	0.071	<0.001	−0.327	−0.093	0.070	0.184	−0.048	−0.799	0.065	<0.001	−0.375
Popularity	0.870	0.104	<0.001	0.339	0.093	0.085	0.277	0.038	−0.083	0.089	0.351	−0.036	0.339	0.077	<0.001	0.131
Liked	0.976	0.304	0.001	0.106	0.773	0.259	0.003	0.088	−0.287	0.240	0.232	−0.035	0.904	0.282	0.001	0.097
Peer-perceived aggression	−0.489	0.191	0.010	−0.088	−0.545	0.148	<0.001	−0.102	−0.134	0.153	0.382	−0.027	−0.288	0.184	0.118	−0.051
*Classroom-level*																
Aggressive descriptive norm	−0.085	0.024	<0.001	−0.599	−0.043	0.028	0.131	−0.701	−0.039	0.026	0.137	−0.347	−0.112	0.030	<0.001	−0.686
Aggressive popularity norm	−0.026	0.085	0.760	−0.062	0.007	0.070	0.924	0.037	0.031	0.062	0.614	0.093	−0.046	0.083	0.585	−0.094
*Residual variance*																
Residual variance within	0.401	0.019	<0.001	0.701	0.442	0.017	<0.001	0.843	0.357	0.019	<0.001	0.768	0.455	0.023	<0.001	0.781
Residual variance between	0.012	0.004	0.001	0.612	0.002	0.003	0.518	0.524	0.011	0.004	0.005	0.893	0.013	0.005	0.018	0.477
Variance explained within	0.299	0.025	<0.001		0.157	0.024	<0.001		0.232	0.030	<0.001		0.219	0.024	<0.001	
Variance explained between	0.388	0.150	0.010		0.476	0.491	0.333		0.107	0.123	0.384		0.523	0.154	0.001	

For academic self-esteem, we controlled for teacher-assigned grade (*B* = 0.250, SE = 0.020, *p* < 0.001, beta = 0.468)

Against expectations (Hypothesis 1b), analyses on descriptive and popularity norms separately indicated that descriptive norms were more strongly related to differences between classrooms in classroom climate perceptions and school adjustment than popularity norms. Beta’s and explained variances for the main models, with descriptive norms and popularity norms being added separately, are reported in Table 4. Beta’s were higher for descriptive norms than for popularity norms. Moreover, descriptive norms explained a higher percentage of the classroom-level variance in classroom climate perceptions and school adjustment when popularity norms were omitted from the model, than vice versa. Interestingly, the significant effects of popularity norms on classroom isolation and conflict even disappeared in models where descriptive norms were omitted. This indicates that the presented effects of aggressive popularity norms in Table 2 should be interpreted with caution given that they were not robust. Consequently, descriptive norms rather than popularity norms predicted variations between classrooms in students’ climate perceptions and adjustment.

**Table 4:** Comparison of beta’s and *R*^2^ for models including descriptive norms and popularity norms separately

	Descriptive norms		Popularity norms	
	Beta	*R*^2^(%)	Beta	*R*^2^(%)
Cooperation	−0.753*	47.0	−0.158	2.5
Conflict	0.789*	53.3	−0.016	2.0
Isolation	0.551*	31.9	−0.085	8.2
Cohesion	−0.569*	30.0	−0.085	4.3
Social self-esteem	−0.620*	38.5	−0.260	6.8
General self-esteem	−0.690*	47.6	−0.185	3.4
Academic self-esteem	−0.315	9.9	−0.020	0.0
Feelings of belonging	−0.718*	51.5	−0.324*	10.5

*p < 0.05

Regarding the covariates included in the main models (Tables 2 and 3), children’s aggression was associated with lower general and social self-esteem and higher perceived isolation. Girls had lower feelings of belonging and lower general and academic self-esteem than boys. Older children perceived more cohesion in their classroom. Students reported more negative climate perceptions in larger classrooms.

### Aggressive Norms and Classroom Climate Perceptions and Adjustment of Victimized, Popular, and Well-liked Students

With regard to students’ social position, main models (Tables 2 and 3) indicated that victims perceived all aspects of the class climate as less positive, had lower feelings of belonging, and had lower general and social self-esteem. Higher popularity was positively associated with perceived cooperation, social self-esteem, and feelings of belonging. Being well-liked was unrelated to students’ classroom climate perceptions but related to stronger feelings of belonging and higher general and social self-esteem.

To test whether the classroom climate perceptions and school adjustment of victims, popular, and well-liked children varied as a function of the classroom aggressive descriptive and popularity norms, cross-level interactions were included between aggressive norms and students’ social status. For the classroom climate outcomes, the AIC indicated a worse fit for models including random slopes of social status and cross-level interactions (M.1A-M.4A compared to M.1C-M.4C; Appendix 1). None of the random slopes varied significantly across classrooms and none of the cross-level interactions were significant (results of models including these random slopes and cross-level interactions are available upon request from first author). This indicates that how a students’ social position was associated with climate perceptions was similar across all classrooms (i.e., no random slopes) and not dependent on either type of norm (no cross-level interactions).

Regarding school adjustment outcomes, the AIC indicated a better fit for the models containing random slopes and cross-level interactions (M.5C-M.8C, Appendix 1) than for the main models. Moreover, multiple random slopes were significant and cross-level interactions explained a considerable proportion of the variance in these slopes (up to 76.7%). Therefore, the models including random slopes and cross-level interactions were interpreted to determine the role of aggressive norms for victimized, popular, and well-liked children’s adjustment. The findings of these analyses are reported in Table 5.

**Table 5:** The role of aggressive peer norms in victimized, well-liked, and popular students’ school adjustment: models containing cross-level interactions

	Social self-esteem			General self-esteem			Academic self-esteem			Belonging		
	*B*	SE	*p*	*B*	SE	*P*	*B*	SE	*p*	*B*	SE	*p*
*Individual-level*												
Gender (0 = boy)	−0.048	0.039	0.223	−0.166	0.045	<0.001	−0.092	0.038	0.015	−0.137	0.044	0.002
Age	−0.005	0.036	0.899	0.004	0.039	0.912	0.001	0.038	0.977	0.004	0.036	0.915
Victimization (0 = non-victim)	−0.582	0.050	<0.001	−0.632	0.073	<0.001	−0.076	0.062	0.225	−0.756	0.056	<0.001
Popularity	0.923	0.098	<0.001	0.134	0.085	0.112	−0.039	0.081	0.627	0.369	0.083	<0.001
Liked	1.102	0.296	<0.001	0.778	0.243	0.001	−0.339	0.234	0.148	0.881	0.285	0.002
Peer-perceived aggression	−0.436	0.202	0.031	−0.619	0.148	<0.001	−0.130	0.159	0.412	−0.352	0.199	0.076
*Classroom-level*												
Aggressive descriptive norm	−0.085	0.023	<0.001	−0.043	0.027	0.110	−0.039	0.026	0.134	−0.113	0.030	<0.001
Aggressive popularity norm	−0.031	0.084	0.712	0.003	0.067	0.946	0.030	0.062	0.632	−0.049	0.083	0.559
*Cross-level interactions*												
Victimization × descriptive norm	−0.072	0.055	0.191	−0.079	0.085	0.353	−0.020	0.069	0.768	−0.143	0.061	0.020
Victimization × popularity norm	−0.313	0.162	0.053	−0.182	0.225	0.418	−0.435	0.151	0.004	−0.491	0.169	0.004
Popularity × descriptive norm	0.034	0.083	0.681	0.083	0.097	0.388	0.051	0.091	0.573	0.051	0.099	0.608
Popularity × popularity norm	−0.661	0.204	0.001	−0.294	0.212	0.165	−0.402	0.182	0.027	−0.241	0.180	0.181
Liked × descriptive norm	0.091	0.292	0.775	−0.022	0.232	0.924	0.326	0.174	0.060	−0.035	0.218	0.871
Liked × popularity norm	1.110	0.774	0.136	1.454	0.556	0.009	0.182	0.628	0.772	0.959	0.765	0.210
*Residual variance*												
Residual variance within	0.382	0.019	<0.001	0.416	0.017	<0.001	0.337	0.018	<0.001	0.437	0.024	<0.001
Residual variance between	0.013	0.004	0.001	0.004	0.003	0.246	0.012	0.004	0.007	0.014	0.006	0.015
Residual variance slope victimization	0.011	0.032	0.721	0.136	0.069	0.049	0.071	0.031	0.020	0.030	0.043	0.478
Residual variance slope popularity	0.116	0.050	0.020	0.053	0.061	0.383	0.048	0.052	0.356	0.041	0.069	0.557
Residual variance slope liked	0.162	0.723	0.822	0.030	10.048	0.977	0.098	0.652	0.881	0.140	0.473	0.767

Covariances among slopes and intercept were modeled but not shown in this table. For academic self-esteem, we controlled for teacher-assigned grade (*B* = 0.247, SE = 0.019, *p* < 0.001)

### Aggressive Norms and Victimized Children

**Fig. 1:**
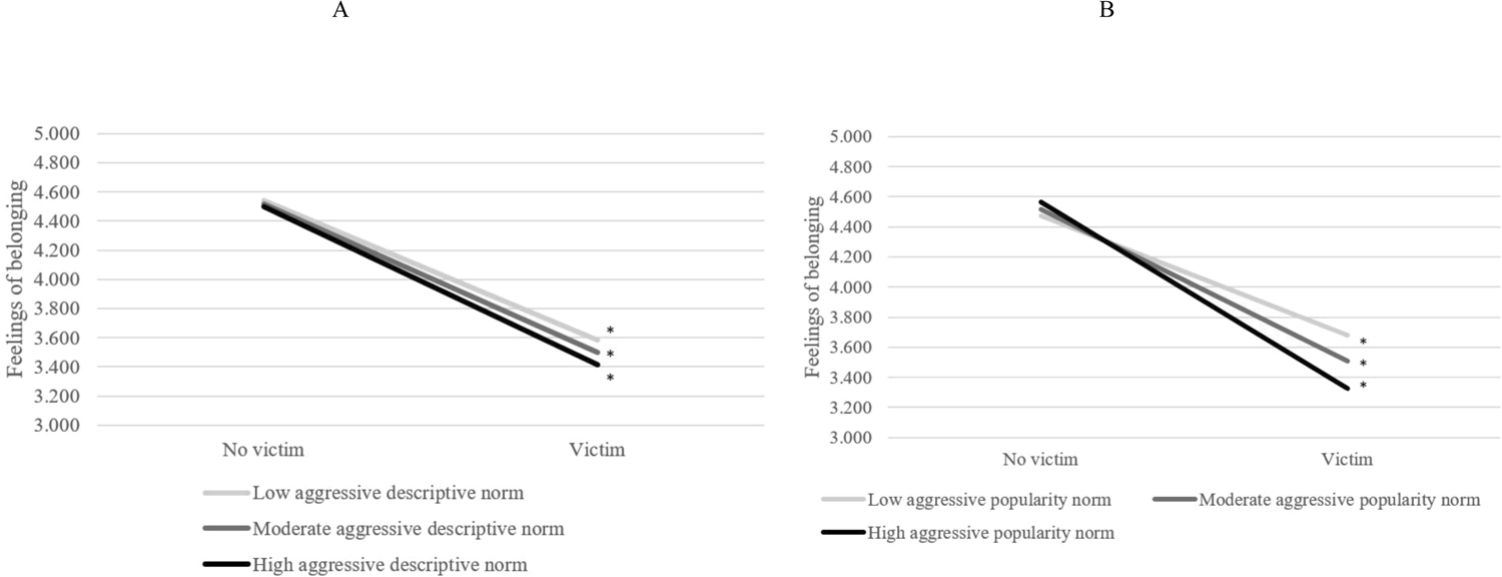
The role of aggressive descriptive norms (**A**) and popularity norms (**B**) in the feelings of belonging of (non-)victimized students. Cut-offs for norms based on 1 SD above and under the mean. Significance of slopes is indicated with an asterisk

In general, victims perceived the classroom climate as more negative and reported lower feelings of belonging as well as lower general and social self-esteem compared to non-victims. There were hardly any cross-level interactions between descriptive norms and students’ victimization. There was one exception, but in an unexpected direction, for victims’ feelings of belonging, *B* = −0.143, SE = 0.061, *p* = 0.020 (Table 5). Figure 1A indicates that victims in classrooms with relatively high aggressive descriptive norms had lower feelings of belonging than victims in classrooms with lower aggressive descriptive norms, which was in contrast to Hypothesis 2a that victims would do better in classrooms with higher aggressive descriptive norms. Moreover, given that there was a main effect of descriptive norms predicting less positive classroom climate perceptions and lower social self-esteem (Tables 2 and 3), and there were no significant cross-level interactions for victims for these outcomes (Table 5), findings indicate that aggressive descriptive norms negatively predicted students’ classroom climate perceptions and social self-esteem, irrespective of whether they were victimized or not. These findings are in contrast to the hypothesis that victimized students would do better in classrooms with higher aggressive descriptive norms (Hypothesis 2a).

Next, in line with Hypothesis 2b, victims’ school maladjustment was found to be exacerbated in classrooms with high aggressive popularity norms, as indicated by three (out of the four tested) cross-level interactions. First, the link between victimization and feelings of belonging varied significantly across classrooms (i.e., random slope), var = 0.129 (0.047), *p* = 0.007. The cross-level interaction with aggressive popularity norms was significant, *B* = −0.491, SE = 0.169, *p* = 0.004 (Table 5). In total 76.7% of the random slope variance was explained, indicating a large effect [Pseudo *R*^2^, calculated as (0.129–0.030)/0.129]. Figure 1B illustrates that victims had lower feelings of belonging in classrooms with high aggressive popularity norms (scoring 1 SD > mean of popularity norms) than in classrooms with low aggressive popularity norms (scoring 1 SD < mean of popularity norms).

**Fig. 2:**
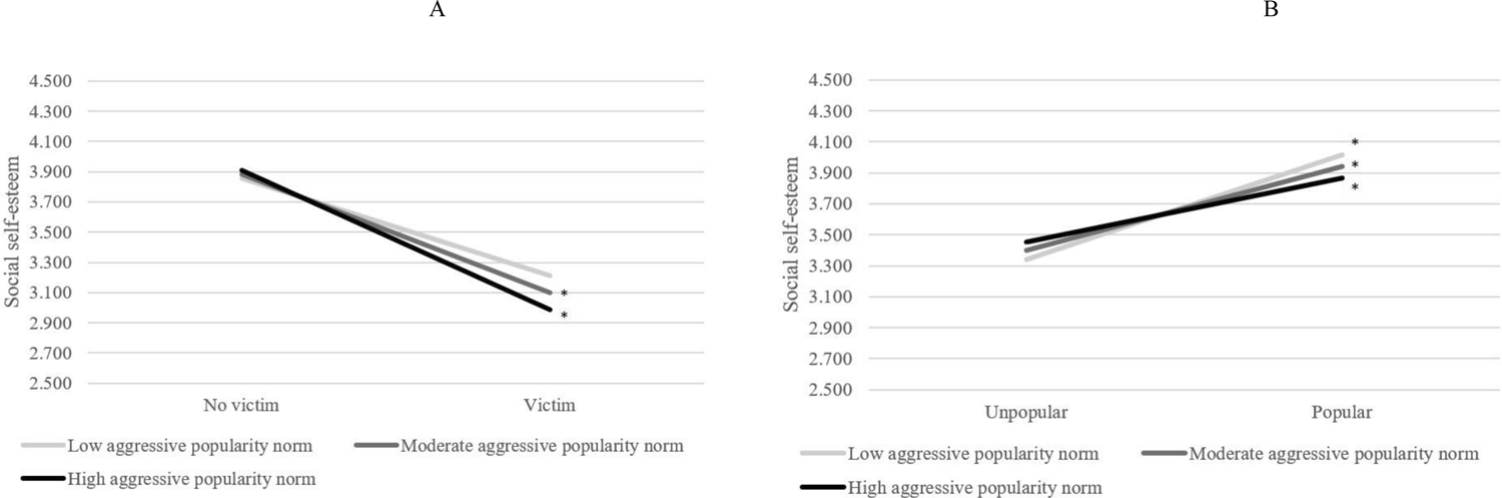
The role of aggressive popularity norms in the social self-esteem of (non-)victimized (**A**) and (un)popular students (**B**). Cut-offs for norms and popular students are based on 1 SD above and under the mean. Significance of slopes is indicated with an asterisk

Second, aggressive popularity norms predicted the random slope of victimization on social self-esteem, *B* = −0.313, SE = 0.162, *p* = 0.053; though this can be considered marginally significant, again a considerable proportion of the variance (75.6%) was explained. Figure 2A illustrates that victims had relatively lower social self-esteem in classrooms with high aggressive popularity norms than victims in classrooms with low aggressive popularity norms.

**Fig. 3:**
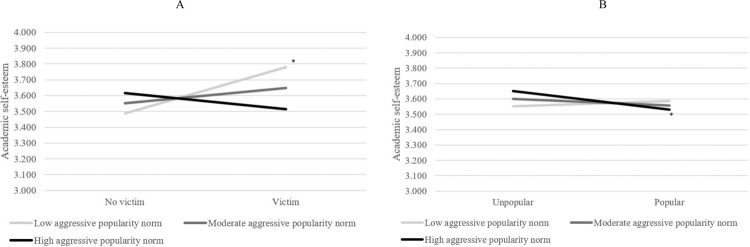
The role of aggressive popularity norms in the academic self-esteem of (non-)victimized (**A**) and (un)popular students (**B**). Cut-offs for norms and popularity are based on 1 SD above and under the mean. Significance of slopes is indicated with an asterisk

Third, the association between victimization and academic self-esteem varied significantly across classrooms, var = 0.110 (0.036), *p* = 0.002. Aggressive popularity norms significantly predicted this random slope variance (*B* = −0.435, SE = 0.151, *p* = 0.004). Aggressive popularity norms explained 35.5% of this variance, indicating a moderate to large effect. Figure 3A shows that victims had a relatively lower academic self-esteem in classrooms with high aggressive popularity norms than victims in classrooms with low aggressive popularity norms. Taken together, these findings of confirmatory analyses are largely in line with Hypothesis 2b that victims would be worse off in classrooms with higher aggressive popularity norms.

### Aggressive Norms and Popular Children

In general, higher popularity was associated with higher perceived cohesion, social self-esteem and feelings of belonging. There were no significant cross-level interactions between descriptive norms and popularity. Together with the significant main effect of descriptive norms (Tables 2, 3, and 5) on classroom climate perceptions, feelings of belonging, and social self-esteem, findings therefore suggest that the role of descriptive norms did not vary depending on students’ popularity. Thus, even though popularity was positively related to individual-level perceived cohesion, social self-esteem, and feelings of belonging, these students were embedded in a context that was, on average, characterized by less positive classroom climate perceptions, social self-esteem, and feelings of belonging. This is in contrast to the hypothesis that popular children would not be bothered by aggressive descriptive norms (Hypothesis 3a).

Regarding popularity norms, there were two significant cross-level interactions that were in line with Hypothesis 3b that popular children would have lower school adjustment in classrooms with higher aggressive popularity norms. First, the slope of popularity on social self-esteem varied significantly across classrooms, var = 0.142, SE = 0.051, *p* = 0.006. This slope was significantly predicted for by the aggressive popularity norm, *B* = −0.661, SE = 0.204, *p* = 0.001. In total, 21.1% of the variance in the slope was explained, indicating a small effect. Figure 2B indicates that popular children (scoring 1 SD above mean of popularity) reported lower social self-esteem in classrooms with higher aggressive popularity norms.

Second, aggressive popularity norms significantly predicted the random slope of popularity on academic self-esteem (*B* = −0.402, SE = 0.182, *p* = 0.027), explaining 11.1% of the variation in this link across classrooms, indicating a small effect. Figure 3B indicates that popular children (scoring 1 SD above mean of popularity) reported lower academic self-esteem in classrooms with aggressive popularity norms than in classrooms with non-aggressive popularity norms. Thus, two out of the four tested findings of confirmatory analyses were in line with the hypothesis that popular children had lower school adjustment in classrooms with higher aggressive popularity norms. This indicates that aggressive popularity norms matter for some but not all aspects of popular students’ adjustment.

### Aggressive Norms and Well-liked Children

**Fig. 4:**
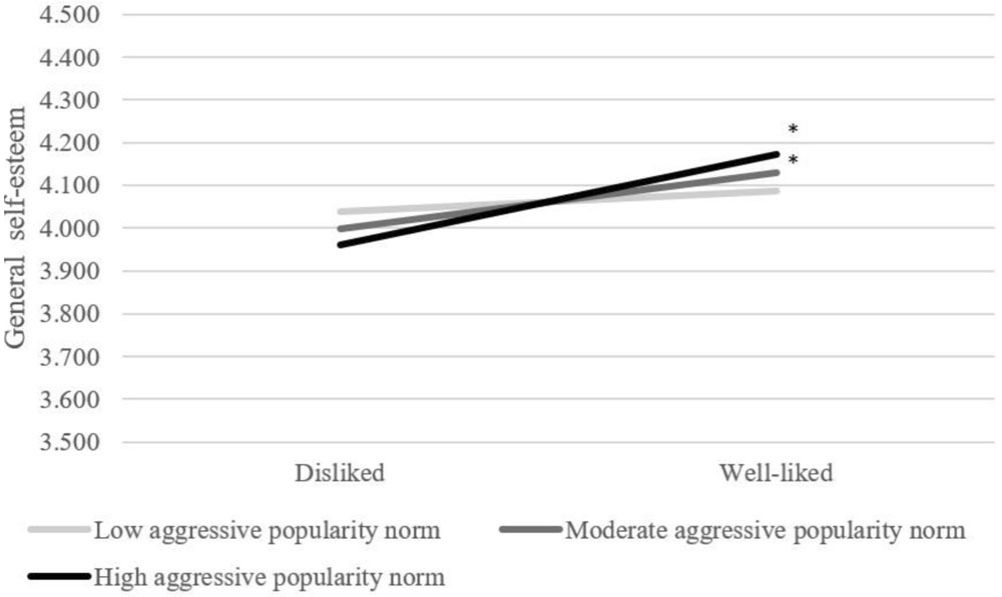
The role of aggressive popularity norms in the general self-esteem of (dis)liked students. Cut-offs for norms and (dis-) liked youth are based on 1 SD above and under the mean. Significance of slopes is indicated with an asterisk

Being well-liked related to higher general and social self-esteem and stronger feelings of belonging (Table 3). It was explored whether descriptive and popularity norms played a role in the classroom climate perceptions and school adjustment of well-liked students. There was only one significant cross-level interaction: The classroom variation of the link between being well-liked and general self-esteem was predicted by aggressive popularity norms (*B* = 1.454, SE = 0.556, *p* = 0.009, Table 5). Figure 4 indicates that well-liked children (>1 SD above the mean of being liked) had higher general self-esteem in classrooms with higher aggressive popularity norms, explaining 23.7% of the variation, indicating a small effect. No cross-level interactions between being well-liked and descriptive norms were detected. Given the main effect of descriptive norms (Tables 2 and 3), findings thus suggest that all students, on average, regarded the classroom climate as less positive when aggressive descriptive norms were higher—irrespective of the extent to which students were well-liked. Thus, being well-liked did not protect against the main effect of aggressive descriptive norms on more negative classroom climate perceptions and various aspects of school adjustment (social self-esteem, and feelings of belonging).

### Additional Analyses

In additional analyses, it was tested whether the classroom climate perceptions and school adjustment of aggressive students varied as a function of aggressive norms. The links between aggression and students’ classroom climate perceptions and school adjustment did not vary significantly between classrooms. Moreover, none of the cross-level interactions with descriptive norms or popularity norms were significant, indicating that the link between aggression and students’ classroom climate perceptions and adjustment did not depend on classroom norms (all *p* varying from 0.098 to 0.883). All other findings presented in the manuscript remained similar after the inclusion of aggression.

Next, additional analyses were run to test interactions between aggressive descriptive and popularity norms, both in predicting main effects and in predicting random slopes. One two-way classroom-level interaction predicting cohesion was significant: classroom cohesion was particularly low in classrooms where descriptive norms were higher and popularity norms were lower (*B* = 0.127, SE = 0.047, *p* = 0.006). Some of the three-way cross-level interactions were significant, too. Specifically, popular peers had lower feelings of belonging and lower social self-esteem in classrooms with both high descriptive and high popularity norms for aggression (*B*_soc_ = 0.314, SE = 0.107, *p* = 0.003; *B*_bel_ = 0.392, SE = 0.104, *p* < 0.001). Victims had lower academic self-esteem when both aggressive descriptive and popularity norms were higher (*B* = 0.185, SE = 0.073, *p* = 0.011).

Potential three-way cross-level interactions with gender and social position on adjustment outcomes were explored (for instance, gender × victimization × popularity norms). None of these three-way cross-level interactions improved model fit or were significant.

### Sensitivity Analyses

Sensitivity analyses were run to test whether results would remain the same when classroom size was omitted as control variable. Indeed, findings were highly comparable. In addition, in analyses in which classrooms’ gender proportion and teacher-assigned grade was controlled for, results remained highly similar and almost no significant effects of these covariates emerged, except that students with a higher teacher-assigned grade perceived lower levels of classroom cohesion (*B* = −0.070, *p* < 0.001). Results of additional and sensitivity analyses can be requested from the first author and are provided on the repository of the Radboud University, Nijmegen, The Netherlands.

## Discussion

In many countries, schools are obliged to formulate protocols that ensure school safety, promote the classroom peer climate, and foster students’ school adjustment. Therefore, the classroom peer climate and students’ school adjustment are important indicators of the quality and effectiveness of schools and are carefully monitored as such by the inspection of education (Orobio de Castro et al., 2018). Peer norms for aggression may hinder schools in achieving their protocol goals. Two types of norms are often distinguished: aggressive descriptive norms reflect how common aggressive behaviors are, and aggressive popularity norms represent the within-classroom correlation between aggression and popularity. Prior work has indicated that popularity norms rather than descriptive norms enhance the acceptance and proliferation of aggression in classrooms (Laninga-Wijnen et al., 2017), but studies parsing out the relative role of these two norms in students’ classroom climate perceptions and school adjustment are lacking. Moreover, norm research primarily focused on adolescents in secondary school, and predominantly considered the role of norms on classrooms in a unified way, without testing whether certain students may be more strongly affected by these norms than others. Therefore, the current study extends upon prior work by identifying the role of descriptive and popularity norms for aggression in perceptions of the classroom climate and school adjustment in elementary school, and by testing whether the role of norms varied depending on students’ social standing (victimization, popularity, and well-liked). Findings indicate that aggressive descriptive and popularity norms had a complementary role: whereas descriptive norms mattered more for the classroom climate as a whole, popularity norms mattered more for the school adjustment of victimized and popular students.

### The Role of Aggressive Norms in Students’ Classroom Climate Perceptions and School Adjustment

In line with Hypothesis 1a, higher aggressive descriptive norms related to less positive classroom climate perceptions as well as lower classroom-levels of social self-esteem and feelings of belonging. This finding aligns with a few prior studies indicating that students in classrooms with high average levels of aggression perceive their classroom as unsafe and full of conflict (Goldstein et al., 2008; Koth et al., 2008), and cared less about their classmates (Gilman et al., 2009). Even though only a small to moderate percentage of the variance in students’ classroom climate perceptions was at the classroom-level (ICC’s varying from 0.086 to 0.169, which is common in multi-level studies, e.g., Huitsing et al., 2019), descriptive norms explained a moderate to large part of this variance (30–53%).

In contrast to Hypothesis 1a, aggressive popularity norms did not predict variations between classrooms in students’ classroom climate perceptions and school adjustment. This was not in line with Hypothesis 1b either, which assumed that especially popularity norms would be important for classroom-level outcomes. Instead, based on findings of the current study two major conclusions can be drawn regarding the role of descriptive and popularity norms in students’ classroom climate perceptions and adjustment (Hypothesis 1b). First, with regard to the classroom climate, descriptive norms seemed to play a more important role than popularity norms. Second, regarding school adjustment, descriptive norms and popularity norms mattered both—but in complementary ways.

Regarding the first conclusion, an important point is that Hypothesis 1b was based on prior work with secondary school students (Dijkstra & Gest, 2015; Laninga-Wijnen et al., 2017). As one of the first to examine the role of popularity norms in relation to student adjustment in elementary school, the current study provides vital insights in the generalizability of previous findings on popularity norms. Even though the desire for popularity may gradually increase in late childhood (Dawes & Xie, 2017), popularity norms do not relate to children’s classroom climate perceptions at this age. It may be that elementary school children are still in an “orientation phase” and explore what popularity means and how they can achieve it. Popularity norms may also matter more in secondary school, due to social and pubertal changes that make adolescents more sensitive to peer influence and motivated to change their reputation in the peer group (Steinberg, 2007; Veenstra & Laninga-Wijnen, 2021). Moreover, there is evidence that children differentiate less between popularity and social preference than adolescents (Van den Berg et al., 2020). This could complicate detecting effects of aggressive popularity norms specifically. Therefore, this study demonstrates the importance of considering developmental differences in parsing out the relative contribution of aggressive descriptive and popularity norms in classrooms.

Regarding the second conclusion on the relative role of norms in school adjustment, the current study found a complementary role for descriptive and popularity norms. Descriptive norms related to lower classroom-levels of social self-esteem and feelings of belonging, possibly because these norms limit students’ opportunities to develop positive relationships with their classmates, which hinders social skill development and decreases feelings of connectedness (Saarento et al., 2015). An important nuance is that—despite its significance and correspondence with other studies (e.g., Huitsing et al., 2019; Schachter & Juvonen, 2018)—the role of descriptive norms in school adjustment was far from substantial. That is, only a small percentage (at most 3.2%) of the variance in children’s school adjustment was at the classroom level. Next, a complementary role of popularity norms was detected: aggressive popularity norms related to more school adjustment problems among popular and victimized youth. The complementary role of descriptive norms and popularity norms for students’ classroom climate perceptions and school adjustment is described into more detail in the paragraphs that follow.

### The Role of Aggressive Norms in the School Adjustment of Victimized, Popular, and Well-liked Children

#### Aggressive norms and victimized children

In contrast to Hypothesis 2a, victimized children did not feel better in classrooms with strong aggressive descriptive norms. Both being victimized and being embedded in classrooms with aggressive descriptive norms contributed to less positive classroom climate perceptions and lower feelings of belonging and social self-esteem, and being victimized did not buffer against the role of norms. This is in contrast to prior studies illuminating a healthy context paradox where victims felt better in classrooms with high levels of victimization (Huitsing et al., 2019) and high levels of teacher-reported aggression (Morrow et al., 2019a). This could be due to how aggressive descriptive norms were assessed in the current study. Aggressive descriptive norms were determined based on the average perceived levels of aggression in classrooms, which does not necessarily imply that there are more victims. It could be that there are many aggressors who all aggress against one victim (i.e., centralization of aggression), but it could also be that everybody is modestly aggressive against each other, and both scenarios may result in comparable levels of aggressive descriptive norms. In line with this reasoning, a prior study indicated the importance of distinguishing between classroom averages of victimization, and centrality of victimization (i.e., classrooms with few victims who are perceived as victims by many classmates) in understanding victims’ adjustment. Classroom-level averages of victimization diminished victims’ plight, whereas centrality of victimization elevated victims’ plight (Huitsing et al., 2012). Future studies are encouraged to parse out the relative role of classroom averages of aggression and centrality of aggression in understanding victims’ adjustment.

In line with Hypothesis 2b, victims had overall a worse school adjustment in classrooms with higher aggressive popularity norms. It is possible that when aggression is endorsed by popular peers, students are less likely to behave against this norm, nor intervene in bullying (Peets et al., 2015)—perhaps because they fear becoming a victim themselves or because they view the aggressive behaviors in a positive light and therefore do not feel the need to intervene. This possibly enhances victims’ feeling that nobody cares about them (Schacter & Juvonen, 2018), and hence, results in enhanced school maladjustment among victims.

#### Aggressive norms and popular children

In contrast to Hypothesis 3a, aggressive descriptive norms predicted lower classroom climate perceptions, feelings of belonging, and social self-esteem, irrespective of students’ popularity. Even though popular students had higher levels of perceived cohesion, social self-esteem, and feelings of belonging compared to non-popular students, these popular students were embedded within a context that was, on average, characterized by less positive classroom climate perceptions, social self-esteem, and feelings of belonging—and being popular did not buffer against the role of these norms. A potential explanation on why being popular may not protect against the role of descriptive norms, is that aggressive classrooms present and unsafe, chaotic environment (Koth et al., 2008), characterized by substantially more negative peer experiences and interactions than less aggressive classrooms. Such negative peer dynamics may make all children perceive their classroom climate as less positive, irrespective of their popularity in the group. This may also be reflected in children’s self-perceptions: they may be more uncertain about their own (social) functioning and feel less connected to their classmates.

In line with Hypothesis 3b, popular students were to some degree less well-adjusted in classrooms with higher aggressive popularity norms. They had lower feelings of belonging and lower academic self-esteem. These findings are in line with prior work indicating that extremely highly popular students may be lonely at the top, perhaps because highly popular students are often highly aggressive (Laninga-Wijnen et al., 2020)—which could make other students to avoid them as potential friends (Ferguson & Ryan, 2019). Also, when popular students are considered as aggressive, their opportunities to collaborate with peers on academic tasks may be limited, which decreases their academic self-esteem.

#### Aggressive norms and well-liked children

Aggressive descriptive norms related to less positive classroom climate perceptions, lower feelings of belonging, and lower social self-esteem among all students, including well-liked students. Well-liked children are often higher on empathy (Oberle et al., 2010)—it therefore can be hypothesized that they are aware of the harmful role of aggressive norms on all students in their classroom, which may make them to perceive their classroom in a negative light. Next, for most outcomes (except general self-esteem) no role was found for aggressive popularity norms in the school adjustment of well-liked students. It thus can be hypothesized that having a supportive social network may buffer against the potential role of aggressive popularity norms but not against the role of aggressive descriptive norms. Perhaps, popular peers may be strategic and selective in their aggression so that they can climb the popularity ladder without losing affection (De Vries et al., 2021; Van der Ploeg et al., 2020), and aggressing against a well-liked peer may be too risky in that regard. Therefore, well-liked youth may be bothered less by aggressive popularity norms.

### Strengths, Limitations, and Future Directions

The current study has several strengths. First, this study is among the first to examine the role of popularity norms in elementary schools, providing new insights in the generalizability of the role of popularity norms previously established for adolescents. Interestingly, it was consistently and robustly found (across varying outcomes) that descriptive norms rather than popularity norms relate to children’s classroom climate perceptions, whereas both descriptive and popularity norms related to students’ school adjustment, in complementary ways. Second, this study extended upon prior work by examining the role of norms for different types of children, indicating that descriptive norms matter for all students, whereas aggressive popularity norms mattered specifically for the school adjustment of popular and victimized children. Third, this study examined the role of aggressive norms in several domains, providing insight in the differential role of norms in a broad array of classroom-level and individual-level outcomes.

Despite these strengths, this study also had limitations. First, due to the design it was only possible to use cross-sectional data, which only allows at assessing concurrent associations. Therefore, direction of effects remains unknown. The current study argued that students perceive aggressive classrooms as less positive due to decreased order and safety and fewer opportunities to establish high-quality peer relationships. Yet, it could also be that students who perceive their classroom as less positive will increase in aggression, for instance due to boredom or as provocative act (Harel-Fisch et al., 2011). This would result in higher aggressive norms. Future longitudinal studies are encouraged to examine the direction of effects.

Second, even though there may be interrelations between different areas of classroom- and student adjustment, convergence issues emerged when outcome variables were included simultaneously, because then there are too many parameters compared to data points. Therefore, models were analyzed for each outcome separately. However, all effects found were in the same direction and clearly demonstrated the lines along which descriptive norms versus popularity norms relate to elementary school children’s perceptions of their classrooms and of themselves.

Third, it is important to acknowledge that victimized, well-liked, and popular students are not three distinct groups of individuals. There may be overlap between them, in particular regarding being well-liked and popular (van den Berg et al., 2020), but also between victimization and popularity (see Dawes & Malamut, 2018). In the current study this overlap was addressed by testing covariances and by including the three types of status simultaneously to control for their relative effects, but an interesting area for future studies would be to explore whether there may be interactions between students’ social positions. For instance, these studies could examine whether victimized popular children would particularly have a lower academic self-esteem in classrooms with higher aggressive popularity norms.

A final limitation is that the sample was relatively homogeneous, consisting of a vast majority of Dutch students, which is partly due to ethnicity being based on country of birth of children and parents (CBS, 2021). Even though the sample’s ethnic composition is in line with ethnic composition of children in The Netherlands (CBS, 2021), it would be interesting to examine whether ethnicity and classroom ethnic composition may be of importance for students’ adjustment (Stevens et al., 2021) or classroom climate perceptions.

Despite these limitations, this study provides important insights in the relative role of descriptive versus popularity norms in elementary schools and questions the generalizability of previous findings for popularity norms in secondary schools (Laninga-Wijnen et al., 2021). High aggressive descriptive norms may signal that each child in these classrooms suffers to a certain extent, and that changes should be brought about. At the same time, in classrooms with aggressive popularity norms, some students are particularly in need of support (victims and popular students). From a developmental perspective, it can be argued that particularly in late childhood or early adolescence, it may be fruitful to conduct peer-led rather than teacher-led interventions. Aggression may be a way to stand up against adult-imposed values, which may be a reason why students are less likely to adhere to rules or regulations provided by teachers (Thomas et al., 2011).

An important question is how this, then, can be established. Various interventions such as the Good Behavior Game aim at promoting the development of non-aggressive, prosocial norms, by motivating (impactful or central) peers to support positive classroom behavior and to take a public stance against aggression (Lannie & McCurdy, 2007). The current study provides a first indication that in elementary schools, a broader implication of this intervention may be needed, by encouraging all students rather than just popular ones to take a public stance against aggression, so that the descriptive norm is targeted. Still, before implications for schools can be derived, future studies are needed to replicate findings over a longer time period and to test whether the direction of effects proposed in this study is correct. Future studies are also encouraged to examine potential buffers against the effects of aggressive norms. For instance, prosocial norms may present a valuable alternative for children to gain access to valuable resources (Ellis et al., 2016). Schools would profit from gaining more insight in the degree to which combinations of aggressive and prosocial norms may contribute to classroom climate experiences and children’s adjustment, especially in elementary education.

## Conclusion

Although prior research has indicated that peer norms for aggression enhance the acceptance and proliferation of aggression in classrooms, it was unclear to date how these norms relate to students’ classroom climate perceptions and school adjustment—and whether this varies across students depending on their social position. The current study indicates that aggressive descriptive norms related to more negative classroom climate perceptions, irrespective of students’ social position. In addition, whereas descriptive norms contributed to between-classroom variations in some aspects of school adjustment, aggressive popularity norms mitigated the school adjustment of some students in particular, namely the victimized and popular children. Thus, whereas work on secondary schools indicated that popularity norms are more important than descriptive norms (e.g., Laninga-Wijnen et al., 2017), the current study demonstrates that in elementary school, both aggressive descriptive and popularity norms matter for students’ school adjustment, in complementary ways.

## Supplementary information

Supplementary Information
