# Supplementary material for: The Role of Aggressive Peer Norms in Elementary School Children’s Perceptions of Classroom Peer Climate and School Adjustment
Source: J Youth Adolesc. 2021 Apr 17;50(8):1582–600. doi: 10.1007/s10964-021-01432-0 (PMC8270866; doi:10.1007/s10964-021-01432-0)
Supplement: Supplementary file 1 — Supplementary Information [file 10964_2021_1432_MOESM1_ESM.docx]

**Appendices**

Appendix 1. Comparisons of main models, models including random slopes, and models including cross-level interactions.

| Model comparisons |  | A  Main Model | B  A + Random slopes | C  B + Cross-level interactions |
| --- | --- | --- | --- | --- |
| Classroom Climate |  |  |  |  |
| M.1 Cooperation |  |  |  |  |
| AIC |  | 3144.809 | 3149.597 | 3146.572 |
| BIC |  | 3255.877 | 3292.398 | 3321.107 |
| u1_vic_ |  |  | 0.055^+^ | 0.027 |
| u2_pop_ |  |  | 0.002 | 0.003 |
| u3_like_ |  |  | 0.266 | 0.172 |
| M.2 Conflict |  |  |  |  |
| AIC |  | 3663.774 | 3672.101 | 3351.612 |
| BIC |  | 3774.841 | 3814.902 | 3504.991 |
| u1_vic_ |  |  | 0.037 | 0.028 |
| u2_pop_ |  |  | 0.016 | 0.013 |
| u3_like_ |  |  | 0.455 | 0.410 |
| M.3 Cohesion |  |  |  |  |
| AIC |  | 3902.510 | 3911.324 | 3680.212 |
| BIC |  | 4013.578 | 4054.125 | 3854.746 |
| u1_vic_ |  |  | .053 | .041 |
| u2_pop_ |  |  | .027 | .021 |
| u3_like_ |  |  | .207 | .209 |
| M.4 Isolation |  |  |  |  |
| AIC |  | 3910.739 | 3922.800 | 3930.604 |
| BIC |  | 4021.806 | 4065.602 | 4105.139 |
| u1_vic_ |  |  | 0.001 | 0.002 |
| u2_pop_ |  |  | 0.020 | 0.020 |
| u3_like_ |  |  | 0.046 | 0.039 |
| School adjustment |  |  |  |  |
| M.5 Belonging |  |  |  |  |
| AIC |  | 3260.159 | 3253.934 | 3245.356 |
| BIC |  | 3344.782 | 3370.291 | 3393.446 |
| u1_vic_ |  |  | 0.129* | 0.030 |
| u2_pop_ |  |  | 0.032 | 0.041 |
| u3_like_ |  |  | 0.220 | 0.140 |
| M. 6 Social self-esteem |  |  |  |  |
| AIC |  | 3077.296 | 3071.450 | 3067.167 |
| BIC |  | 3161.919 | 3187.807 | 3215.257 |
| u1_vic_ |  |  | 0.045 | 0.011 |
| u2_pop_ |  |  | 0.142** | 0.116* |
| u3_like_ |  |  | 0.190 | 0.162 |
| M. 7 Academic self-esteem^$^ |  |  |  |  |
| AIC |  | 2696.415 | 2686.114 | 2686.460 |
| BIC |  | 2784.999 | 2805.963 | 2837.574 |
| u1_vic_ |  |  | 0.110** | 0.071* |
| u2_pop_ |  |  | 0.054 | 0.048 |
| u3_like_ |  |  | 0.136 | 0.098 |
| M.8 General self-esteem |  |  |  |  |
| AIC |  | 3192.964 | 3180.883 | 3184.024 |
| BIC |  | 3277.587 | 3297.240 | 3332.114 |
| u1_vic_ |  |  | 0.173** | .136 |
| u2_pop_ |  |  | 0.063 | .053 |
| u3_like_ |  |  | 0.039 | .030 |

*Note.* ^$^ For academic self-esteem, students’ teacher-assigned grade was controlled for in the models. The gray models are selected based on pre-defined criteria, and hence, presented and interpreted in the manuscript.
